# People learn a two-stage control for faster locomotor interception

**DOI:** 10.1007/s00426-023-01826-8

**Published:** 2023-04-21

**Authors:** Huaiyong Zhao, Dominik Straub, Constantin A. Rothkopf

**Affiliations:** 1 Department of Psychology, Zhejiang Sci-Tech University, Hangzhou, China; 2 Institute of Psychology, Technical University of Darmstadt, Darmstadt, Germany; 3 Centre for Cognitive Science, Technical University of Darmstadt, Darmstadt, Germany; 4 Frankfurt Institute for Advanced Studies, Goethe University, Frankfurt, Germany

## Abstract

People can use the constant target-heading (CTH) strategy or the constant bearing (CB) strategy to guide their locomotor interception. But it is still unclear whether people can learn new interception behavior. Here, we investigated how people learn to adjust their steering to intercept targets faster. Participants steered a car to intercept a moving target in a virtual environment similar to a natural open field. Their baseline interceptions were better accounted for by the CTH strategy. After five learning sessions across multiple days, in which participants received feedback about their interception durations, they adopted a two-stage control: a quick initial burst of turning accompanied by an increase of the target-heading angle during early interception was followed by significantly less turning with small changes in target-heading angle during late interception. The target’s bearing angle did not only show this two-stage pattern but also changed comparatively little during late interception, leaving it unclear which strategy participants had adopted. In a following test session, the two-stage pattern of participants’ turning adjustment and the target-heading angle transferred to new target conditions and a new environment without visual information about an allocentric reference frame, which should preclude participants from using the CB strategy. Indeed, the pattern of the target’s bearing angle did not transfer to all the new conditions. These results suggest that participants learned a two-stage control for faster interception: they learned to quickly increase the target-heading angle during early interception and subsequently follow the CTH strategy during late interception.

## Introduction

Locomotor interception of a moving target is a fundamental behavior that is widely performed by animals (e.g., Ghose, Horiuchi, Krishnaprasad, & Moss, 2006; Lanchester & Mark, 1975; Olberg, Worthington, & Venator, 2000;) and humans (e.g., Chapman, 1968; McBeath et al., 1995; Mcleod & Dienes, 1996). Research in this field has identified a couple of interception strategies, which individually have been able to describe participants' interceptive behavior across different experimental paradigms. However, much less is known about actors’ learning behavior in locomotor interception. In daily lives, people often need to learn new action skills. Can people adapt their interceptive behavior and learn a new interception control? We ran the current study to answer this question.

### Locomotor interception strategies

**Fig. 1:**
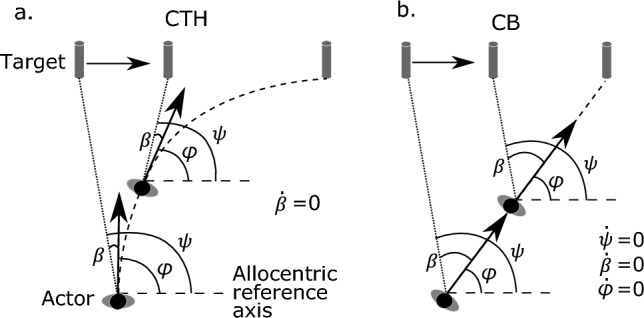
Schematics of the constant target-heading (CTH) strategy (**a**) and the constant bearing (CB) strategy (**b**) in an interception scenario in which both the actor and the target move at constant speeds. Both the heading angle of the actor (*φ*) and the bearing angle of the moving target (*ψ*) are defined relative to an allocentric reference direction. The target-heading angle (*β*) is defined as the angle between these two angles (*β* = *ψ* – *φ*). The associated constraints of the two strategies are listed in each panel, respectively

Two prominent strategies have been proposed to explain the guidance of locomotor interception when both human actors and the target move on the same horizontal plane (Fig. 1). Actors using the constant bearing (CB) strategy keep the target at a constant angle relative to an allocentric reference axis during interception. Therefore, this strategy depends on the availability of information about an allocentric reference frame. In theory, the CB strategy produces linear interception paths with a constant heading direction. By contrast, actors using the constant target-heading (CTH) strategy keep the target at a constant angle relative to the current heading direction. Accordingly, this strategy does not depend on the availability of information about allocentric reference frames; instead, it requires only the information about the target’s direction and the actor’s heading direction. In theory, because the CTH strategy only constraints the rate of change of the target-heading angle to be zero, different constant values of the target-heading angle across individual participants and trials are possible. Therefore, this strategy can produce both linear and curved interception paths, where the degree of curvature depends on the specific value of the target-heading angle. In a special case of the CTH strategy, the value of the target-heading angle is kept at zero throughout the interception, which indicates that the actor is pursuing the target.

Several studies on locomotor interception have provided evidence for the CTH strategy and the CB strategy in experimental paradigms involving speed control tasks. In such a task, actors are only able to control their speed along a fixed straight path to intercept a target that moves toward their path (Bastin et al., 2006, 2008; Chardenon et al., 2002, 2004, 2005; Lenoir et al., 1999, 2002). Because of this specific task constraint, the target’s bearing angle always covaries with the target-heading angle during interception. Therefore, studies involving this experimental paradigm cannot discriminate between the two strategies.

A few studies have examined participants’ interception strategy in steering tasks, in which the participants control the direction of interception movement. For example, Rushton et al. (1998) showed that participants walked directly toward the target’s current location during interception with the target-heading angle close to zero. Their results suggest that people use the CTH strategy to guide interception of a slow target (e.g., about 1°/s at the beginning of a trial in Rushton et al., 1998). Later, Fajen and Warren (2004) showed that participants walked in a direction ahead of the current location of a faster target (about 8°/s at the beginning of a trial) during interception. A similar result was also reported by Bootsma and colleagues who asked participants to control their direction of motion using a steering wheel to intercept a moving target in virtual environments (Casanova et al., 2022; Ceyte et al., 2021). However, no clear behavioral evidence for any particular interception strategy, such as small changes in either the target-heading angle or the target’s bearing angle, was reported in these three studies. One possible reason for the absence of clear behavioral evidence may be that interception durations in these studies were comparatively short (e.g., it was 3 or 4 s in Casanova, et al., 2022). Short interception durations might not allow participants to bring any of the angular variables to a constant value before they completed the interception.

Zhao, Straub, and Rothkopf (2019) directly investigated people’s strategy for locomotor interception with a longer duration. They asked participants to steer a car to intercept a moving target in virtual environments. The task was designed so that participants’ interception durations spanned from about 6 to12 s. Using this task, the authors found clear evidence that participants kept the target at a constant target-heading angle throughout the interception, consistent with the CTH strategy. By contrast, the target’s bearing angle continuously changed during interception, inconsistent with the CB strategy.

Moreover, using a similar interception task, Zhao, Straub, and Rothkopf (2021) showed that the CTH strategy was used in different virtual environments which varied in the amount of visual information about allocentric reference frames. First, in this study, different participants consistently steered interception paths with different degrees of curvature, from curved ones to linear ones. Second, all participants maintained an approximately constant target-heading angle during the later period of interception (i.e., the last 40% of an interception course) with its absolute rate of change significantly lower than that of the target’s bearing angle. Finally, one particular participant consistently steered linear interception paths, with little change in the target’s bearing angle, even in the environment without any visual information about allocentric reference frames. Since the CB strategy depends on the availability of information about an allocentric reference frame, this participant’s interception control could not be attributed to the CB strategy. Taken together, the study found convincing evidence that all these interception behaviors were better explained by the CTH strategy with consistent individual differences between participants in the magnitude of the target-heading angle, leading to interception paths with different degrees of curvature.

If we define interception efficiency according to the interception path length, which in the case of participants’ constant velocity corresponds to efficiency in the duration of the interception, participants’ different interception paths in Zhao et al. (2021) reflect different degrees of efficiency. Specifically, curved interception paths correspond to relatively longer paths and durations, which is less efficient; linear interception paths correspond to relatively shorter paths and durations, which is more efficient. Thus, in the current study, we examined, whether participants can learn a new and more efficient interception control and furthermore, whether they can generalize the learned control to new conditions.

### Development and learning of interceptive locomotion

While the literature on skill acquisition and learning of visuomotor behaviors in motor control (Krakauer et al., 2019) and sports science (Hodges & Williams, 2012) is extensive, much less is known about the development and learning of interceptive locomotion behavior. Improvement in performance on manual and locomotor tasks is widely observed in children and adults. It has been suggested that better performance in these tasks may be due to detection of and change in the information that people use to guide action at different stages of practice (e.g., Fajen & Devaney, 2006; Fajen, 2008a, 2008b; Jacobs & Michaels, 2006; Smith et al., 2001; van Hof et al., 2006). The developmental changes in coordinating self and object movement in school-aged children have been investigated in locomotor interception tasks (Chihak et al., 2010, 2014; Chohan, Verheul, van Kempen, Wind, & Savelsberg, 2008). Chohan et al. (2008) examined younger and older children’s speed control as they walked along a fixed straight path to intercept a real moving object. Though all children successfully intercepted the target, younger children (aged 5–7 years) had difficulties effectively coupling their speed with the target’s speed, resulting in their speed control deviating from the CTH or the CB strategy.^1^ In contrast, older children (aged 10–12 years) effectively coupled their speed with the target’s speed, consistent with the CTH strategy.

Chilhad et al. (2010) showed that children (age 10 and 12 years) were less effective than adults as they rode a bicycle to pass through a gap between two blocks, which successively moved along a virtual roadway. Specifically, children had less time to spare than adults in this gap-interception task, and they approached the gap with a more pronounced corrective speed adjustment. In this study, neither children’s nor adults’ speed adjustment was consistent with the CTH strategy, probably because participants were not familiar with such a task, and they had a small number of practice and test interceptions. Specifically, they performed only a single interception as practice and subsequently 12 interceptions as the test.

Later, Chihak et al. (2014) examined children’s and adults’ learning in this gap-interception task. This study showed that several interception trials could not help children acquire a consistent interception strategy. Specifically, their corrective speed adjustment appeared still more pronounced and variable between different interception conditions (i.e., either speeding up or slowing down was required for interception). In contrast, after adults initially had performed several interceptions with pronounced corrective speed adjustment, they showed a consistent pattern of speed adjustment which was consistent with the CTH strategy. The abovementioned findings suggest that the CTH strategy may have been acquired by adult participants and that a few practice trials can lead to using it.

### The current study

We previously reported that most of the participants steered curved interception paths while a couple of them steered apparently linear interception paths in an open virtual environment (Zhao, et al., 2021). The different patterns of interception paths may be closely related to different interception efficiency. In the current study, we examined whether adult participants can learn to change their interception behavior, specifically, steering more efficient linear interception paths. We additionally investigated, in case participants learned to adjust their interception behavior, whether the learned behavior generalizes to new conditions. Moreover, we asked whether participants leaned to adopt a different interception strategy, such as the CB strategy.

To investigate these questions, we asked participants to steer a car to intercept a target moving at different speeds across conditions in an open virtual environment, which provided visual information about allocentric reference frames. Participants were instructed to intercept the target accurately and to try to intercept it as fast as possible. After extensive learning of this task in this environment, participants were tested with new target conditions in both the environment used during learning and in a new environment, which did not provide visual information about allocentric reference frames. If participants successfully learned to use the CB strategy based on a visual allocentric reference frame, the learned pattern of interceptive steering should generalize to the new target conditions in the environment used during learning, but be absent in the environment without access to an allocentric reference frame. Though it has been reported that the proprioceptive information from maneuvers of the steering wheel alone is not sufficient to accurately maintain a stable allocentric reference frame (Wallis, Chatziastros, &Bülthoff, 2002; Wallis et al., 2007), it might become sufficient through extensive learning of the task. Therefore, if participants successfully learned to use the CB strategy based on the proprioceptive information about allocentric reference frames, the learned pattern of interceptive steering should be observed in all target conditions in both the learning environment and the new environment.

## Methods

### Participants

Ten students at the Technical University of Darmstadt participated in the experiment, two of which dropped out before completing the experiment and were therefore excluded from data analysis. Thus, eight participants finished all six experimental sessions (three women, five men; *M*_age_ = 24.6 years, *SD*_age_ = 4.7 years). All participants had a driver’s license for at least three years but no previous experience performing an interception task in a virtual environment. Some participants wore glasses for corrected vision during the task. Before the experiment, participants signed an informed consent form; after the experiment, they received payment or participation credit from the Institute of Psychology. All experimental procedures are approved by the ethics committee of the Technical University of Darmstadt.

### Apparatus

We used the same apparatus as in Zhao et al., (2019, 2021). We used Vizard (WorldViz, Santa Barbara, CA, US) to generate all the virtual environments. The display was presented in a head-mounted display (HMD, Oculus Rift DK 2, OculusVR, Irvine, CA, US) at 60 Hz, which provided stereoscopic viewing with an 80° (vertical) × 80° (horizontal) field of view. Participants sat on a chair and used a force feedback steering wheel with a turning range of 450° left/right (Driving Force GT, Logitech, Newark, CA, US) fixed to a desk in front of the chair to control the heading direction. The steering wheel’s current turning angle determined the car’s current turning rate, with each 5° of turning angle mapping to 1°/s turning rate. The car’s heading was updated by integrating the turning rate of the car on each frame as follows:

1 $$\varphi_{i + 1} = \varphi + \Delta t_{i} \dot{\varphi }_{i} ,$$

where ${\varphi }_{i}$ is the heading direction in the *i*th frame, ${\dot{\varphi }}_{i}$ is the turning rate, and $\Delta {t}_{i}$ is the duration of that frame, respectively. The car’s location was updated by translating it in its current heading direction at a fixed speed of 7 m/s.

### Display

**Fig. 2:**
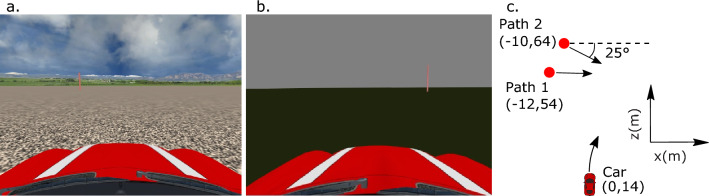
Participant’s view of the virtual environment and schematic of the experimental setup. The textured-ground environment (**a**) and the green-ground environment (**b**) are shown as seen by a participant during the experiment. The layout of the initial locations of the car and the target in the x–z plane are shown in a top-down view (**c**)

Participants performed the interception task in two virtual environments also used in Zhao et al. (2021) (see Fig. 2). The *textured-ground* environment was designed as an open field with rich visual information about allocentric reference frames: it consisted of a round ground plane with a radius of 300 m and a random noise texture, a blue sky with clouds, and a surrounding background landscape at the edge of the ground plane. By contrast, the *green-ground* environment was designed without the visual information about allocentric reference frames: it consisted of only a uniformly gray sky and a textureless green ground plane with the same size as that in the first environment. In both environments, the target was a solid red cylinder, which was 3 m tall with a radius of 0.2 m. The car was 3.6 m long, 1.8 m wide, and 1.5 m tall. To specify the locations of the objects in the environments, we set the center of the ground plane as the origin of the environments (x = 0, z = 0 m). To prevent the persistent influence of landmarks across trials on interception in the textured-ground environment, the surrounding background image and the sky/clouds were rotated randomly by an angle from 0° to 270° about the origin of the environments before the start of each trial.

The car appeared at the origin (x = 0, z = 0 m) at the beginning of each trial, facing the positive z direction, and moving straight ahead along a green guidance strip (10.5 m long) on the ground. After the car moved 14 m in the positive z direction, the target appeared and moved along one of two paths (Fig. 2). Along Path 1, the target appeared at 40 m ahead and 12 m to the left of the car (x = –12, z = 54 m, i.e., 16.7° to the left relative to the z-axis), and moved rightward on the path parallel to the x-axis. Along Path 2, the target appeared at 50 m ahead and 10 m to the left of the car (x =  – 10, z = 64 m, i.e., 11.3° to the left relative to the *z*-axis), and approached the car at an angle of 25° to the *x*-axis. Participants steered the car to intercept the target. The initial location and moving direction of the target were mirrored left/right about the *z*-axis in different trials, and data were collapsed in the analysis. A trial ended when the center of the car was within a distance of 0.8 m to the target center or if the car went 1 m further than the target in the positive z direction (i.e., the participant missed the target.)

### Design and procedure

Throughout all conditions of the experiment, participants were instructed to intercept the target as accurately and quickly as possible. We used a within-subjects factorial design (see Table 1). First, we examined whether participants could learn to change their interception behavior through five learning sessions on five different days, respectively. Each of these sessions consisted of two blocks, resulting in 10 learning blocks in total. The first learning block was to examine participants’ baseline performance; participants received no feedback about the duration of their interception. From the second learning block on, participants received feedback about their interception duration on each trial. This allowed participants to adjust their interceptions in order to achieve shorter paths and durations. In each of the blocks, the target moved at 4.5 or 5.5 m/s always along Path 1 in the textured-ground environment. Each target speed was repeated 24 times, which gave rise to 48 trials presented in random order in each of the learning blocks.

**Table 1:** Design of the experiment

	5 learning sessions	1 test session
Blocks × trials	10 × 48	2 (environments) × 80
Feedback	None in Block 1, available in Blocks 2-10	None
Target speed	4.5, 5.5	4.0, 4.5, 5.0, 5.5, 6.0
Target path	Path 1	Path 1, path 2
Environment	Textured-ground	Textured-ground, then green-ground

The unit of target speed is m/s. In the test session, interceptions in the first block were always in the textured-ground environment and those in the second block were always in the green-ground environment.

Second, to investigate whether participants had learned a new interception strategy, we examined whether the learned behavior generalized to new conditions. The test session was conducted about 2 days after the last learning session (*M* = 2.13, *SD* = 1.96 days). It consisted of two blocks without any feedback about participants’ interception duration. The first test block was to examine whether the learned behavior generalized to new target speeds and paths. In this block, participants performed interceptions always in the textured-ground environment. The target moved along either Path 1 or Path 2, at speeds of 4.0, 4.5, 5.0, 5.5, or 6.0 m/s, which gave rise to 10 speed-by-path conditions. Each of these 10 target conditions was repeated 8 times, which gave rise to 80 trials presented in random order in this block. The second test block was to examine whether the learned behavior generalized to a new environment, specifically the green-ground environment. In this block, participants performed 80 trials presented in random order, as in the first test block, but in the green-ground environment. There were two reasons for the fixed sequence of the two test blocks: first, we wanted to avoid the influence of the new environment on the test of the influence of the new target speeds and path in the old environment; second, we considered interceptions in the new environment as a further test relative to those in the old environment.

Before the first learning block, participants performed 12 interceptions as practice with the same conditions as in the learning block. Each learning session lasted for about 50 min; each test session lasted for about 100 min. Participants were debriefed about their performance after the experiment.

### Data analysis

Since the car was the end effector, we used the car’s position and orientation for data analysis. We defined a trial as successful if the target came within a radius of half a car length (1.8 m) of the car’s center. Interception location was defined as the location where the car reached its minimum distance to the target. Interception duration spanned from the moment the target appeared to the end of the trial.

The car’s heading direction relative to the x-axis was recorded on each frame (φ_i_ on the ith frame). The corresponding target’s bearing angle relative to the x-axis was computed according to the following equation:

2 $$\psi_{{\text{i}}} = {\text{ arccot }}\left[ {\left( {{\text{X}}_{{\text{i}}} {-}{\text{ x}}_{{\text{i}}} } \right) \, / \, \left( {{\text{Z}}_{{\text{i}}} {-}{\text{ z}}_{{\text{i}}} } \right)} \right]$$

Then the target-heading angle was computed as β_i_ = ψ_i_ – φ_i_. To compute the absolute rate of change of these angular variables, we divided their absolute difference between two successive frames by the duration between the two frames, respectively.

When participants came close to the target toward the end of interception, the short distance between the car and the target usually caused quick changes in both the target’s bearing and the target-heading angles. To eliminate these artifacts, we truncated each trial at the point in time when the center of the car reached a distance of 3.6 m to the target as in previous experiments on locomotor interception (e.g., see Lenoir, et al., 1999). Then, the normalized mean time series of all angular variables of interest were computed. First, the raw data points of each trial were divided into 50 bins with an approximately equal number of data points in each bin. Second, the first data point in each bin was set as the value of the bin. Finally, the mean in each bin was computed across trials for each participant to yield a normalized mean time series for each condition. A similar data analysis was used in Zhao et al., (2019, 2021).

## Results

### The learning sessions

We examined whether learning occurred throughout the learning sessions. Participants completed 3840 learning trials in total and the target was only missed on four out of all the trials. This clearly indicates that participants were able to intercept the target successfully on most of the trials. Because of the very low rate of misses, the failed trials were considered exceptions and removed from our data analysis. Which strategy could better describe participants’ interception is determined by examining the absolute rate of change of the TH and bearing angles as suggested by the two strategies, the CB and CTH strategy, according to Fig. 1.

**Fig. 3:**
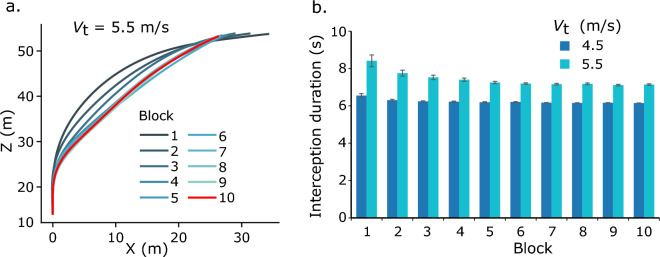
Exemplary interception paths (**a**) and overall mean interception duration (**b**). The mean interception paths from a representative participant are shown with different colors for different learning blocks. The mean interception durations across all participants are shown together with error bars representing the ± *SE*

*Interception duration.* First, we examined how participants’ interceptions changed through learning by analyzing their interception paths across different blocks. Figure 3a shows a representative participant’s mean interception paths in the 10 learning blocks, with one path for each block. The interception paths appear to become more linear and shorter through learning. The paths of the last several blocks overlap one another, implying that performance in the last several blocks reached a ceiling. The pattern of participants’ interception paths was confirmed by our analysis of interception duration (Fig. 3b). To examine the learning effect on interception duration, we ran a two-way repeated-measures analysis of variance (ANOVA, 2 target speeds × 4 learning blocks) on interception duration in the first two and the last two learning blocks. Note that, in the first learning block feedback about interception duration was not provided to participants, and it was provided from the second learning block on. The results indicated a significant main effect of target speed, *F*(1, 7) = 179.30, *p* < 0.01, *η*_p_^2^ = 0.96, a significant main effect of learning block, *F*(3, 21) = 17.59, *p* < 0.01, *η*_p_^2^ = 0.72, and a significant interaction between them *F*(3, 21) = 22.19, *p* < 0.01, *η*_p_^2^ = 0.76. Post hoc pairwise comparisons with Sidak adjustment revealed similar results for both the slow (4.5 m/s) and the fast (5.5 m/s) target. Specifically, neither the difference in the interception durations between the first two learning blocks nor the difference between the last two learning blocks was significant (*p* > 0.05). In contrast, the interception duration in any one of the last two learning blocks was significantly shorter than that of any one of the first two learning blocks (*p* < 0.05). The results show that participants intercepted the target with shorter duration through learning. Additionally, their interceptions in the last two blocks took similar durations, implying that their interception performance may have reached a ceiling. To gain a better understanding of the effects of learning on participants’ interceptions, we examined the first and the last learning blocks in the following analyses.

**Fig. 4:**
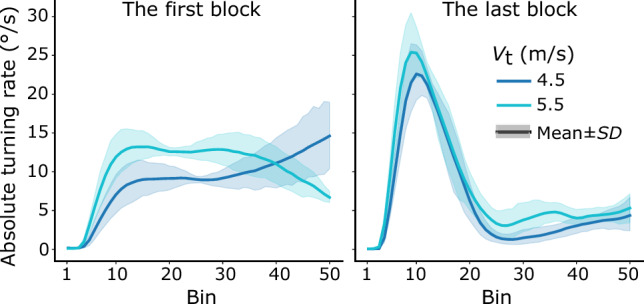
Mean time series of absolute turning rate in the first (left) and the last (right) learning blocks over all participants

*Turning rate.* To further examine how participants changed their interceptive steering through learning, we analyzed their absolute turning rate. Absolute turning rate reflects the curvature of an interception path: higher absolute turning rates lead to more curved interception paths, whereas a turning rate of zero leads to a straight interception path. Figure 4a shows that in the first learning block, participants’ absolute turning rates increased apparently at the beginning of interception, then stayed approximately constant for at least half of the interception duration, and subsequently increased or decreased gradually toward the end of interception. In the last learning block, by contrast, Fig. 4b shows an apparent peak-then-flat pattern, i.e., the mean absolute turning rate rose and then dropped quickly in the first half of the interception period and afterwards remained at relatively low values below 5°/s in the second half of the interception period. To closely examine participants’ absolute turning rate, we divided an interception course into the early and the late periods. The early period was defined as the first 40% of the interception course (the first 20 data points of the normalized time series), the late period the last 40% (the last 20 data points of the normalized time series), with the middle 20% as a transition period for a clear differentiation between the early and the late periods. The mean absolute turning rates of the early and the late periods were computed by averaging the data points over the two periods, respectively.

A three-way repeated-measure ANOVA (2 interception periods × 2 learning blocks × 2 target speeds) on the mean absolute turning rates indicated a significant main effect of interception period, *F*(1, 7) = 5.81, *p* < *0.05*, *η*_p_^2^ = 0.45, a significant main effect of target speed, *F*(1, 7) = 20.43, *p* < *0.01*, *η*_p_^2^ = 0.75, a significant three-way interaction of the factors, *F*(1, 7) = 14.58, *p* < *0.01*, *η*_p_^2^ = 0.68, but no significant main effect of learning block, *F*(1, 7) = 3.94, *p* > *0.05*. We then examined whether there was any significant difference in the mean absolute turning rates between the early and the late interception periods and whether this changed through learning. A follow-up simple effect test with Sidak adjustment indicated that, in the first learning block, the mean absolute turning rates were not significantly different between the early and the late periods, for either target speed (*F* < 5.51, *p* > 0.05); however, in the last learning block, the mean absolute turning rates in the late period were significantly lower than those in the early period, for both target speed (*F* > 317.57, *p* < 0.01). The results indicate that, at the beginning of learning, participants steered the car with comparable turning adjustment during the early and late interception periods; at the end of learning, in contrast, relatively larger turning adjustment occurred in the early period, and smaller turning adjustment occurred in the late interception period (see Fig. 4).

We additionally examined how participants’ absolute turning rates changed through learning in the two interception periods, respectively. For the early interception period, a two-way repeated-measures ANOVA (2 target speeds × 2 learning blocks) on the mean absolute turning rates indicated a significant main effect of target speed, *F*(1, 7) = 319.55, *p* < 0.01, *η*_p_^2^ = 0.98, a significant main effect of learning block, *F*(1, 7) = 65.34, *p* < 0.01, *η*_p_^2^ = 0.90, and a significant interaction between them *F*(1, 7) = 25.25, *p* < 0.01, *η*_p_^2^ = 0.78. A follow-up simple effect test with Sidak adjustment revealed a significant main effect of learning block for both target speeds of 4.5 m/s, *F*(1, 7) = 59.49, *p* < 0.01 and 5.5 m/s, *F*(1, 7) = 72.83, *p* < 0.01. These results provide evidence for a clear learning effect, i.e. a significant increase of the mean absolute turning rates in the early interception period between the first and the last learning block.

For the late interception period, the same analysis indicated no significant main effect of target speed, *F*(1, 7) = 0.16, *p* > 0.05, but a significant main effect of learning block, *F*(1, 7) = 417.95, *p* < 0.01, *η*_p_^2^ = 0.83, and a significant interaction between them *F*(1, 7) = 9.02, *p* < 0.05, *η*_p_^2^ = 0.56. A follow-up simple effect test with Sidak adjustment revealed a significant main effect of learning block for both target speed of 4.5 m/s, *F*(1, 7) = 29.63, *p* < 0.01 and 5.5 m/s, *F*(1, 7) = 38.57, *p* < 0.01. The results also indicated a clear learning effect, albeit in the opposite direction compared to the early interception period, i.e. the mean absolute turning rates significantly decreased in the late interception period from the first to the last learning block.

In summary, the main results of the analyses of participants’ absolute turning rates were as follows. Before leaning, participants’ mean absolute turning rates were comparable between the early and the late interception periods. Through learning, participants’ mean absolute turning rates increased in the early interception period but decreased in the late interception period. This pattern of steering led to significantly larger and then smaller turning adjustment in the early and the late interception periods, respectively.

**Fig. 5:**
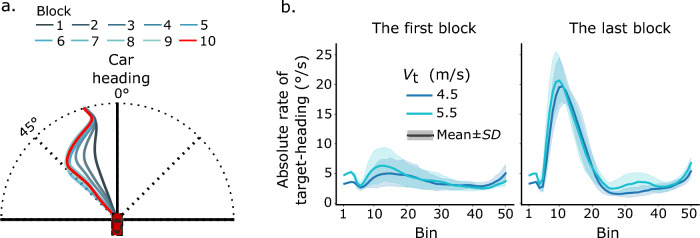
The mean time series of the target-heading angle across the 10 learning blocks resulting from the steering control of a typical participant (**a**), and the difference in the mean time series of the absolute rate of change of the target-heading angle over all participants (**b**) with the first block (left) and last block (right) of trials

*The target-heading angle.* To better understand the steering pattern in terms of possible interception strategies, we analyzed whether participants’ interceptions were consistent with the CTH strategy by analyzing the changes in target-heading angle. Figure 5a shows one representative participant’s mean time series of the target-heading angle in the 10 learning blocks, respectively. Across the learning blocks, the average target-heading angle changed apparently in the early interception period, while almost no changes were identifiable during the late interception period. Figure 5b shows the mean time series of absolute rates of the target-heading angle for all participants. In the first learning block, the absolute rates of the target-heading angle increased at the beginning of interception, then decreased gradually. In the last learning block, by contrast, we observed the peak-then-flat pattern again, i.e., the absolute rates rose (at about 20°/s) and then dropped quickly in the early interception period and subsequently stayed at relatively low values in the late interception period. Therefore, we followed the analysis of the mean absolute turning rates and analyzed the mean absolute rates of the target-heading angle over the early and late interception periods.

A three-way repeated-measure ANOVA (2 interception periods × 2 learning blocks × 2 target speeds) indicated a significant main effect of interception period, *F*(1, 7) = 160.71, *p* < *0.01*, *η*_p_^2^ = 0.96, a significant main effect of learning block, *F*(1, 7) = 52.48, *p* < *0.01*, *η*_p_^2^ = 0.88, a significant main effect of target speed, *F*(1, 7) = 13.40, *p* < *0.01*, *η*_p_^2^ = 0.66, and a significant three-way interaction of the factors, *F*(1, 7) = 16.18, *p* < *0.01*, *η*_p_^2^ = 0.69. We then examined whether there was any significant difference in the mean absolute rates between the early and late interception periods and whether this changed through learning. A follow-up simple effect test with Sidak adjustment indicated that the mean absolute rates in the late interception period were significantly lower than those in the early interception period, across all target speeds and learning blocks (2 target speeds by 2 learning blocks; for them all, *F* > 5.85, *p* < 0.05). The results suggest that significantly lower absolute rates of the target-heading angle in the late interception period may be a general and stable pattern of participants’ interceptive steering (see Fig. 5b).

We additionally examined how the mean absolute rates of the target-heading angle changed through learning within the two different interception periods, respectively. For the early interception period, a two-way repeated-measures ANOVA (2 target speeds × 2 learning blocks) indicated a significant main effect of target speed, *F*(1, 7) = 10.82, *p* < *0.05*, *η*_p_^2^ = 0.61, a significant main effect of learning block, *F*(1, 7) = 110.08, *p* < *0.01*, *η*_p_^2^ = 0.94, but no significant interaction between them, *F*(1, 7) = 3.93, *p* > 0.05. These results indicated a clear learning effect, i.e., that the mean absolute rates of the target-heading angle in the early interception period significantly increased from the first to the last learning block. Moreover, in the last learning block, the peaks of the absolute rates of the target-heading angle reached about 20°/s early during interception (see Fig. 5b), which is apparently not consistent with the CTH strategy.

For the late interception period, the same analysis indicated no significant main effect of target speed, *F*(1, 7) = 2.67, *p* = *0.15*, no significant main effect of learning block, *F*(1, 7) = 0.01, *p* = *0.94*, but a significant interaction between them, *F*(1, 7) = 13.81, *p* < 0.01, *η*_p_^2^ = 0.66. A follow-up simple effect test with Sidak adjustment revealed no significant main effect of learning block for either target speed of 4.5 m/s, *F*(1, 7) = 1.95, *p* = 0.21. or 5.5 m/s, *F*(1, 7) = 1.72, *p* = 0.23. Low mean absolute rates of the target-heading angle were observed in the late interception periods across different target speeds and learning blocks. The lowest mean absolute rate was observed for target speed of 4.5 m/s in the last learning block (*M* = 2.71°/s, *SD* = 0.99°/s); the highest was observed for target speed of 5.5 m/s in the last learning block (*M* = 3.60°/s, *SD* = 1.32°/s). The results suggest a stable pattern of target-heading angle across different conditions, i.e., during the late interception period participants steered in a way that the target-heading angle changed less than a few degrees per second.

To summarize the main results of the analyses of the absolute rates of the target-heading angle: The mean absolute rates significantly increased in the early interception period through learning, but these rates remained at low values and changed little during the late interception period across the learning blocks. Moreover, the mean absolute rates in the late interception period were always significantly lower than those in the early interception period, which was a general pattern observed across different conditions throughout learning.

**Fig. 6:**
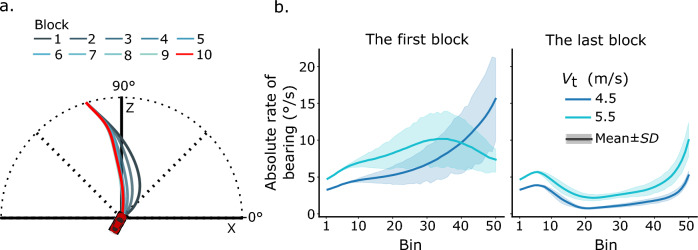
The mean time series of the target’s bearing angle across the 10 learning blocks resulting from the steering control of a typical participant (**a**), and the difference in the mean time series of the absolute rate of change of the bearing angle over all participants (**b**), specifically the first block (left) and the last block (right)

*The target’s bearing angle.* Following the above analysis, we analyzed the target’s bearing angle to test whether participants’ steering is consistent with the CB strategy. Figure 6a shows the mean time series of the target’s bearing angle of one representative participant for the 10 learning blocks, respectively. The bearing angle did change in the first learning block, but it changed less so in the later blocks.

A three-way repeated-measure ANOVA (2 interception periods × 2 learning blocks × 2 target speeds) on the mean absolute rates of the target’s bearing angle indicated a significant main effect of interception period, *F*(1, 7) = 5.95, *p* < *0.05*, *η*_p_^2^ = 0.46, a significant main effect of learning block, *F*(1, 7) = 17.44, *p* < *0.01*, *η*_p_^2^ = 0.71, a significant main effect of target speed, *F*(1, 7) = 35.63, *p* < *0.01*, *η*_p_^2^ = 0.84, and a significant three-way interaction of the factors, *F*(1, 7) = 13.57, *p* < *0.01*, *η*_p_^2^ = 0.66. We then examined whether there was any significant difference in the mean absolute rates between the early and late interception periods and whether this changed through the learning blocks. A follow-up simple effect test with Sidak adjustment indicated that the mean absolute rates in the late interception period were significantly higher than those in the early interception period for target speed of 4.5 m/s in the first learning block, *F* = 7.84, *p* < 0.05; the mean absolute rates were not significantly different between different periods in other speed-by-block conditions (for them all, *F* < 5.02, *p* > 0.05). In the first learning block, there was no common pattern of the mean absolute rates of the target’s bearing angle over the two interception periods, i.e., they were significantly different for the slow target, but they were not so for the fast target; in the last learning block, in contrast, a common pattern emerged across different target speeds, i.e., the mean absolute rates were not significantly different between the two interception periods (see Fig. 6b).

In accordance with the analysis of the target-heading angle, we closely examined how the mean absolute rates of the target’s bearing angle changed through learning in the two different interception periods, respectively. For the early interception period, a two-way repeated-measures ANOVA (2 target speeds × 2 learning blocks) indicated a significant main effect of target speed, *F*(1, 7) = 146.39, *p* < 0.01, *η*_p_^2^ = 0.95, a significant main effect of learning block, *F*(1, 7) = 104.94, *p* < 0.01, *η*_p_^2^ = 0.94, and a significant interaction between them, *F*(1, 7) = 57.94, *p* < 0.01, *η*_p_^2^ = 0.89. A follow-up simple effect test with Sidak adjustment revealed a significant main effect of learning block for both target speeds of 4.5 m/s, *F*(1, 7) = 114.36, *p* < 0.01 and 5.5 m/s, *F*(1, 7) = 97.68, *p* < 0.01. The results indicated a clear effect of learning, i.e., the mean absolute rates of the target’s bearing angle in the early interception period significantly decreased from the first to the last learning block.

For the late interception period, the same analysis indicated no significant main effect of target speed, *F*(1, 7) = 1.59, *p* = *0.25*, but a significant main effect of learning block, *F*(1, 7) = 11.76, *p* < 0.05, *η*_p_^2^ = 0.63, and a significant interaction between them *F*(1, 7) = 10.49, *p* < 0.05, *η*_p_^2^ = 0.60. A follow-up simple effect test with Sidak adjustment revealed a significant main effect of learning block for both target speeds of 4.5 m/s, *F*(1, 7) = 12.06, *p* = 0.01 and 5.5 m/s, *F*(1, 7) = 10.65, *p* < 0.05. The results also indicated a clear learning effect, i.e. the mean absolute rates of the target’s bearing angle in the late interception period significantly decreased through learning (Fig. 6b).

Taken together, the main results regarding the absolute rates of change of the target’s bearing can be summarized as follows: Through learning, the mean absolute rates of the target’s bearing angle significantly decreased in both the early and the late interception periods. In the last learning block, a common pattern emerged for both the slow and fast targets, i.e., the mean absolute rates of the target’s bearing angle were not significantly different between the early and late interception periods.

*The target-heading angle vs. the target’s bearing angle in the late interception period.* To examine which strategy, the CB or the CTH, could better describe participants’ interceptions, we compared the mean absolute rates of change of the two angles in the late interception period. In the first learning block with the slow target (4.5 m/s) the mean absolute rates of the target-heading angle and the target’s bearing angle were 3.29°/s (*SD* = 1.02°/s) and 10.28°/s (*SD* = 6.49°/s), respectively; for the fast target (5.5 m/s), they were 2.97°/s (*SD* = 0.87°/s) and 9.22°/s (*SD* = 4.71°/s), respectively. Two paired *t-*tests indicated that the mean absolute rates of the target-heading angle were significantly lower than those of the target’s bearing angle, for both the slow target, *t*(7) = 2.69, *p* < 0.05, *d* = 0.95, and the fast target, *t*(7) = 3.20, *p* < 0.05, *d* = 1.13. This result suggests that, before the learning blocks, participants’ interceptive steering was more consistent with the CTH strategy.

In the last learning block, the mean absolute rates of the target-heading angle and the target’s bearing angle for the slow target were 2.71°/s (*SD* = 0.99°/s) and 2.17°/s (*SD* = 0.47°/s), respectively; for the fast target, they were 3.60°/s (*SD* = 1.32°/s) and 4.51°/s (*SD* = 1.57°/s), respectively. Two paired *t*-tests indicated that the mean absolute rates of the two angles were not significantly different from each other for either the slow target, *t*(7) =  − 1.72, *p* > 0.05, or the fast target, *t*(7) = 1.18, *p* > 0.05. The results indicated that the mean absolute rates of the two angles were comparable in the late interception period in the last learning block. Thus, only on the basis of the rates of change in the two angles, we could not differentiate between the CTH and the CB strategy in this block. Based on this result, it could be possible that participants had learned to use the CB strategy to guide their interceptions.

### The test session

Participants completed 1280 test trials in total, during which they missed the target only on five of these trials. The failed trials were then removed from the following analysis. On these remaining trials, we tested whether participants could generalize the learned pattern of steering to new target speeds and new target paths in the old (the textured-ground) environment, and furthermore, to the new (the green-ground) environment. We computed the mean absolute rates of change of the angles over the early and late interception periods, as we did for the learning blocks.

*Interception duration.* In the test session, participants completed interceptions with durations ranging from about 6 s to 9 s across all conditions (Table 2). A trend is apparent that interception duration increased with the target speed across all the four environment-by-path conditions. The difference in interception duration between the slowest and the fastest targets was about 2 to 3 s for Path 1 across different environments. In contrast, the difference was about 0.5 to 1.5 s for Path 2 across different environments. The relatively smaller difference in the latter case may be due to the fact that the targets approached participants along Path 2 (see Fig. 2).

**Fig. 7:**
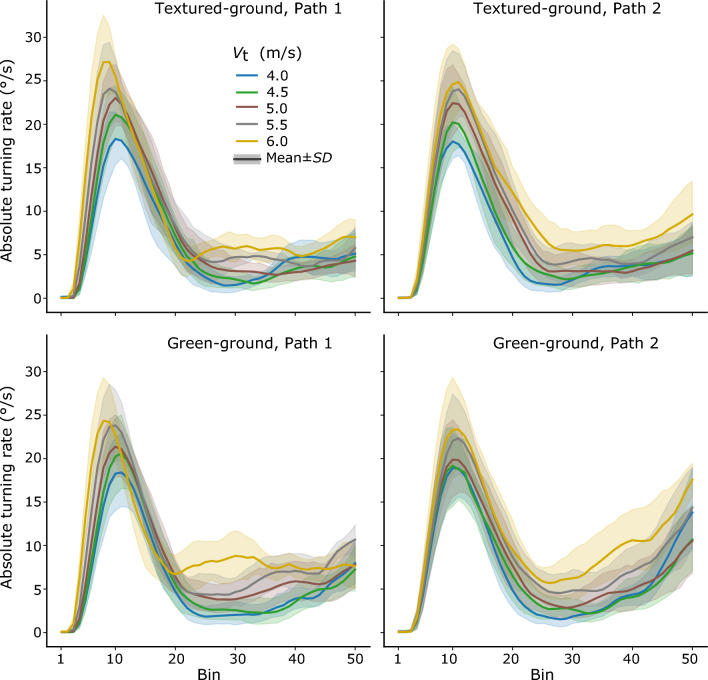
The mean time series of absolute turning rate over all participants for different velocities of the target. Top row: textured ground environment path 1 (left) and path 2 (right). Bottom row: green-ground environment path 1 (left) and path 2 (right)

*Turning rate*. Figure 7 shows the mean time series of absolute turning rate. Compared with the peak-then-flat pattern of the absolute turning rates in the last learning block (see Fig. 4b), in the test session, the peak part with a large increase followed by a large decrease in the turning rate appeared well maintained in all conditions, whereas the flat part did not appear to be well maintained in some conditions. We examined whether the learned pattern of turning adjustment transferred to the test session, i.e., whether relatively larger turning adjustment occurred in the early interception period and smaller turning adjustment in the late interception period. In the textured-ground environment, a three-way repeated-measures ANOVA (2 interception periods × 2 target paths × 5 target speeds) on the mean absolute turning rates indicated a significant main effect of interception period, *F*(1, 7) = 117.56, *p* < *0.01, η*_p_^2^ = 0.94, a significant main effect of target speed, *F*(4, 28) = 52.48, *p* < *0.01, η*_p_^2^ = 0.88, and a significant interaction between them, *F*(4, 28) = 13.66, *p* < *0.01, η*_p_^2^ = 0.66, but no significant main effect of target path, *F*(1, 7) = 0.082, *p* = *0.78,* or related significant interaction involving target path (for them all, *p* > 0.21). A follow-up simple effect test, across target paths with Sidak adjustment, indicated that the mean absolute turning rates in the late interception period were significantly lower than those in the early interception period for all the five target speeds (*F* > 73.79, *p* < 0.01, for them all). The results indicated significantly larger and then smaller turning adjustment in the early and then late interception periods, respectively; the learned pattern of turning adjustment held for the new target speeds and target path in the old environment (see the upper panels of Fig. 7).

**Table 2:** Mean interception duration and *SD* across participants in the test session

Conditions	Textured-ground		Green-ground	
	Path1	Path2	Path1	Path2
V_t_ 4	5.94 (0.03)	5.92 (0.02)	5.95 (0.05)	5.99 (0.06)
V_t_ 4.5	6.16 (0.03)	5.90 (0.05)	6.20 (0.11)	5.95 (0.13)
V_t_ 5	6.52 (0.05)	5.92 (0.04)	6.63 (0.19)	6.00 (0.16)
V_t_ 5.5	7.17 (0.18)	6.01 (0.06)	7.36 (0.31)	6.18 (0.27)
V_t_ 6	8.44 (0.42)	6.24 (0.15)	9.10 (0.78)	6.65 (0.65)

The unity of interception duration is second

In the green-ground environment, the same analysis indicated significant main effects of interception period, *F*(1, 7) = 12.64, *p* < *0.01, η*_p_^2^ = 0.64, target path, *F*(1, 7) = 9.16, *p* < *0.05, η*_p_^2^ = 0.57, and target speed, *F*(1.37, 9.55) = 31.37, *p* < *0.01, η*_p_^2^ = 0.82 (with Greenhouse–Geisser adjustment), and a significant interaction between target path and speed, *F*(4, 28) = 4.55, *p* < *0.01, η*_p_^2^ = 0.39. These results indicated significantly larger and then smaller turning adjustment in the early and then late interception periods, respectively; the learned pattern of turning adjustment also transferred to the new environment (see the lower panels of Fig. 7).

**Fig. 8:**
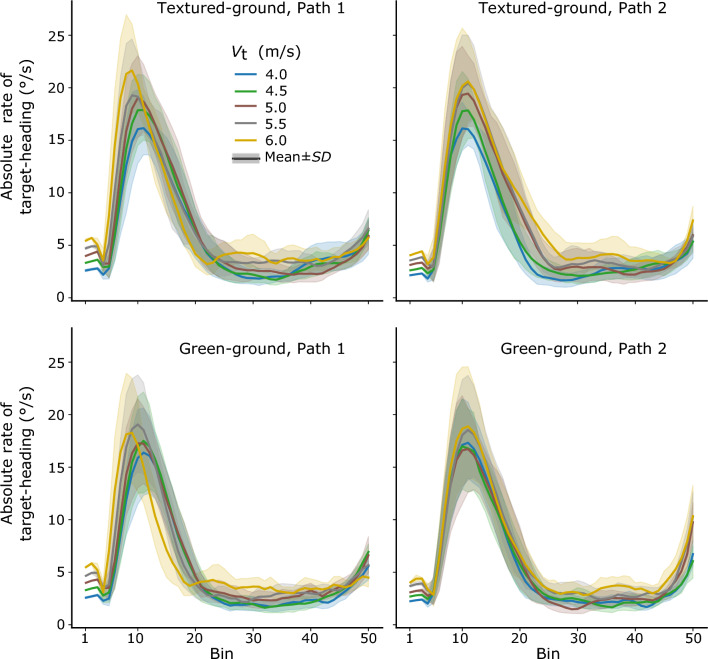
The mean time series of absolute rate of change of the target-heading angle over all participants for different velocities of the target. Top row: textured ground environment path 1 (left) and path 2 (right). Bottom row: green-ground environment path 1 (left) and path 2 (right)

*The target-heading angle.* Figure 8 shows the mean time series of the absolute rate of the target-heading angle in different conditions. Note that the peak-then-flat pattern is present across different conditions, similar to that in the last learning block. We examined whether the general pattern of the absolute rate of the target-heading angle transferred to the different conditions in the test session, i.e., whether they showed significantly higher and then lower absolute rates of the target-heading angle in the early and then late interception period, respectively. In the textured-ground environment, a three-way repeated-measures ANOVA (2 interception periods × 2 target paths × 5 target speeds) indicated significant main effects of interception period, *F*(1, 7) = 182.08, *p* < *0.01, η*_p_^2^ = 0.96, target speed, *F*(1.30, 9.12) = 16.29, *p* < *0.01, η*_p_^2^ = 0.69 (with Greenhouse–Geisser adjustment), a significant interaction between interception period and target speed, *F*(4, 28) = 8.46, *p* < *0.01, η*_p_^2^ = 0.55, and a significant interaction between target path and speed, *F*(4, 28) = 2.72, *p* < *0.05, η*_p_^2^ = 0.28; other main effect and interactions were not significant (*p* > 0.05). A follow-up simple effect test, across target paths with Sidak adjustment, indicated that the mean absolute rates of the target-heading angle in the late interception period were significantly lower than those in the early interception period, for all the five target speeds (*F* > 81.48, *p* < 0.01, for them all). These results suggest that the general pattern of the target-heading angle held for the new target speeds and the new path in the old environment (see the upper panels of Fig. 8).

In the green-ground environment, the same analysis indicated significant main effects of interception period, *F*(1, 7) = 62.43, *p* < *0.01, η*_p_^2^ = 0.89, target path, *F*(1, 7) = 10.01, *p* < *0.05, η*_p_^2^ = 0.59, and target speed, *F*(4, 28) = 11.45, *p* < *0.01, η*_p_^2^ = 0.62, and a significant interaction between target path and speed, *F*(4, 28) = 6.29, *p* < *0.01, η*_p_^2^ = 0.47. There was no significant interaction involving the interception period. The significant main effect of the interception period indicated that the mean absolute rates of the target-heading angle in the late interception period were significantly lower than those in the early interception period. The results suggest that the general pattern of the target heading angle transferred to the new environment, implying the pattern of the target-heading angle during interception was stable across conditions (see the lower panels of Fig. 8).

**Fig. 9:**
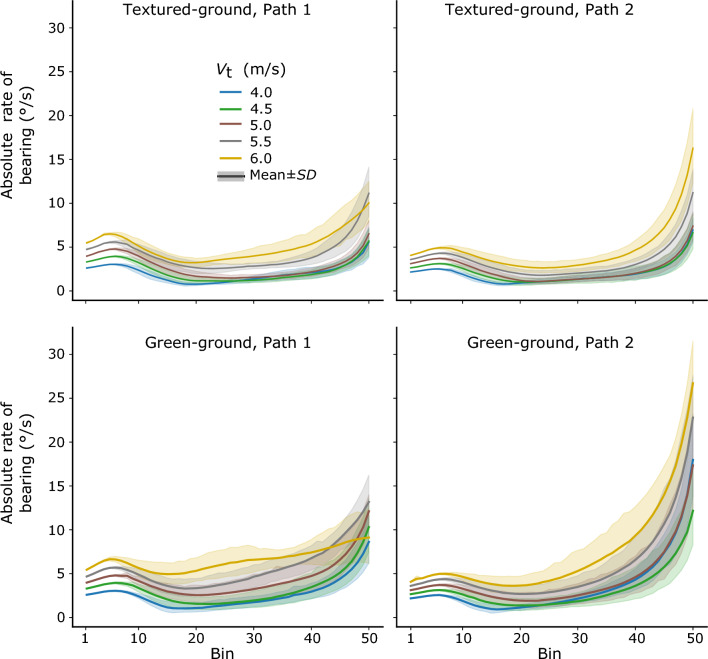
The mean time series of absolute rate of change of the target’s bearing angle over all participants for different velocities of the target. Top row: textured ground environment path 1 (left) and path 2 (right). Bottom row: green-ground environment path 1 (left) and path 2 (right)

*Target’s bearing angle.* We examined whether comparable mean absolute rates of change of the target’s bearing angle in the early and late interception periods transferred to the test session. In the textured-ground environment, a three-way repeated-measures ANOVA (2 interception periods × 2 target paths × 5 target speeds) on the mean absolute rates indicated significant main effects of target path, *F*(1, 7) = 7.77, *p* < *0.05, η*_p_^2^ = 0.53, and target speed, *F*(1.56, 10.89) = 45.68, *p* < *0.01, η*_p_^2^ = 0.87 (with Greenhouse–Geisser adjustment); the main effect of interception period was not significant, *F*(1, 7) = 3.25, *p* > *0.05,* but the interaction between interception period and target speed was significant, *F*(4, 28) = 6.08, *p* < *0.01, η*_p_^2^ = 0.47. A follow-up simple effect test across target paths with Sidak adjustment indicated that the mean absolute rates of the target’s bearing angle in the late interception period were significantly higher than those in the early interception period for target speed 4.0 m/s (*F* = *9.36*, *p* < 0.05), and 6.0 m/s (*F* = *5.98*, *p* < 0.05); but, they were not significantly different between each other for other target speeds (*F* < *3.15*, *p* > 0.12, for them all). The results indicated that the learned pattern of the target’s bearing angle did not hold for the slowest and the fastest targets in the old environment (see the upper panels of Fig. 9).

In the green-ground environment, the same analysis indicated significant main effects of interception period, *F*(1, 7) = 26.86, *p* < *0.01, η*_p_^2^ = 0.79, and target speed, *F*(4, 28) = 32.43, *p* < *0.01, η*_p_^2^ = 0.82, and a significant interaction between interception period and target speed, *F*(4, 28) = 4.673.61, *p* < *0.01, η*_p_^2^ = 0.40, a significant interaction between interception period and target path, *F*(1, 7) = 13.40, *p* < *0.01, η*_p_^2^ = 0.66. The main effect of target path was not significant, *F*(1, 7) = 2.28, *p* > *0.05.* A follow-up simple effect test with Sidak adjustment indicated that the mean absolute rates of the target’s bearing angle in the late interception period were significantly higher than those in the early interception period in 7 out of the 10 target speed-by-path conditions, but they were not significantly different between each other in the remaining three target conditions (see Table 3). The results indicated that the learned pattern of the target’s bearing angle held in some target conditions, but did not hold in other target conditions in the new environment (see the lower panels of Fig. 9).

**Table 3:** F-values for the follow-up simple effect test of the absolute rate of the target’s bearing angle in the green-ground environment in the test session

	Path1	Path2
V_t_ 4	8.96*	25.76**
V_t_ 4.5	3.29	5.17
V_t_ 5	6.75*	6.83*
V_t_ 5.5	33.50**	15.39**
V_t_ 6	5.09	35.44**

V_t_ = target speed; * *p* < 0.05; ** *p* < 0.01

*Target-heading angle vs. target’s bearing angle in the late interception period.* In the last learning block, the mean absolute rates of the target-heading angle and the target’s bearing angle during the late interception period were not significantly different between each other. Here, we examined whether this result transferred to the test session.

For the target-heading angle in the textured-ground environment, the lowest mean absolute rate in the late interception period was observed in the condition with target speed of 5 m/s on Path 1, *M* = 2.95°/s, *SD* = 0.58°/s; the highest in the condition with target speed of 6 m/s on path 2, *M* = 4.01°/s, *SD* = 1.04°/s. In the green-ground environment, the lowest mean absolute rate was observed in the condition with target speed of 4.5 m/s on Path 2, *M* = 2.61°/s, *SD* = 0.55°/s; the highest in the condition with target speed of 6 m/s on path 2, *M* = 4.24°/s, *SD* = 0.86°/s.

In comparison, for the target’s bearing angle in the textured-ground environment, the lowest mean absolute rate in the late interception period was observed in the condition with target speed of 4.5 m/s on Path 1, *M* = 2.35°/s, *SD* = 0.61°/s; the highest in the condition with target speed of 6 m/s on path 2, *M* = 6.17°/s, *SD* = 2.38°/s. In the green-ground environment, the lowest mean absolute rate was observed in the condition with target speed of 4 m/s on Path 1, *M* = 3.68°/s, *SD* = 1.81°/s; the highest in the condition with target speed of 6 m/s on path 2, *M* = 11.59°/s, *SD* = 4.17°/s.

We compared the mean absolute rates of the two angles in the late interception period in each condition. The *t* values of the paired comparisons are listed in Table 4. In the textured-ground environment, the mean absolute rates of the target-heading angle and the target’s bearing angle were not significantly different in 8 out of 10 target conditions, but the former was significantly lower than the latter in two of the target conditions. In the green-ground environment, the mean absolute rates of the target-heading angle and the target’s bearing angle were not significantly different in three out of 10 target conditions, but the former was significantly lower than the latter in 7 of the target conditions. Taken together, these results suggest that comparable mean absolute rates of the two angles did not strictly hold in either environment. The overall comparisons imply that the CTH strategy is more consistent with participants’ interceptions in both the old and the new environments in the test session. Note that this is the same conclusion drawn for participants’ baseline performance in the first learning block before their extensive learning.

**Table 4:** *t*-values for paired comparison between target-heading and target’s bearing in the late interception period in the test session

Conditions	Textured-ground		Green-ground	
	Path1	Path2	Path1	Path2
V_t_ 4	1.91	0.76	− 2.41*	− 3.99**
V_t_ 4.5	2.02	1.36	− 1.79	− 1.91
V_t_ 5	0.72	0.75	− 2.44*	− 2.23
V_t_ 5.5	− 1.58	− 1.41	− 5.88**	− 3.47*
V_t_ 6	− 2.69 *	− 2.45*	− 4.50**	− 5.02**

V_t_ = target speed; * *p* < 0.05; ** *p* < 0.01

*Target-heading angle in the early interception period.* Although the absolute rate of the target-heading angle remained quite low during the late interception period, it rose and dropped quickly in the early interception period. Such a quick rise and drop apparently is not consistent with the CTH strategy. To gain a better understanding of this steering behavior, we examined whether this rise-then-fall pattern, i.e., the change in mean absolute rate of the target-heading angle in the early interception period, was maintained across different conditions or whether it varied across different conditions.

In the textured-ground environment, a two-way repeated-measures ANOVA (2 target paths × 5 target speeds) on the mean absolute rates of the target-heading angle indicated no significant main effect of target path, *F*(1, 7) = 0.071, *p* > *0.05,* but a significant main effect of target speed, *F*(1.47, 10.29) = 16.39, *p* < *0.01, η*_p_^2^ = 0.70 (with Greenhouse–Geisser adjustment), and a significant interaction between them, *F*(4, 28) = 4.69, *p* < *0.01, η*_p_^2^ = 0.40. In the green-ground environment, the same analysis indicated a significant main effect of target path, *F*(1, 7) = 7.84, *p* < *0.05, η*_p_^2^ = 0.53, a significant main effect of target speed, *F*(1.93, 13.53) = 3.93, *p* < *0.05, η*_p_^2^ = 0.36 (with Greenhouse–Geisser adjustment), and a significant interaction between them, *F*(4, 28) = 4.84, *p* < *0.01, η*_p_^2^ = 0.41. These results indicated that the absolute rate of the target-heading angle in the early interception period varied across different conditions, instead of being a constant stereotyped pattern.

Finally, as the CTH strategy does not prescribe a particular value for the target-heading angle, we analyzed how its value changed between its initial value when the target appeared within the scene and its final value at interception. The initial target-heading angle was 16.69° for target Path 1 and 11.31° for Path 2, respectively. Mainly due to the pronounced increase of the angle in the early interception period, the final target-heading angle across different conditions generally ranged from approximately 30° to 40°. Moreover, the between-participants *SD* and the mean within-participants *SD* appear quite low (see Table 5). This implies the consistency in the pattern of interceptive steering both within and between participants.

**Table 5:** Mean final target-heading angle followed by its between-participants *SD* and its mean within-participants *SD*

Conditions	Textured-ground		Green-ground	
	Path1	Path2	Path1	Path2
V_t_ 4	32.66 (2.42, 4.84)	28.26 (2.89, 4.06)	33.06 (3.52, 6.96)	32.33 (6.11, 8.14)
V_t_ 4.5	36.07 (2.15, 3.97)	30.86 (1.64, 4.51)	32.56 (4.10, 6.73)	29.69 (6.22, 7.07)
V_t_ 5	40.00 (2.36, 4.22)	34.78 (1.56, 4.16)	35.31 (6.04, 6.41)	28.35 (6.15, 6.36)
V_t_ 5.5	40.04 (4.27, 5.43)	37.65 (3.06, 4.17)	36.31 (5.53, 5.85)	30.24 (6.08, 7.78)
V_t_ 6	44.01 (6.44, 4.17)	40.28 (4.39, 5.06)	38.81 (8.10, 6.69)	30.93 (6.58, 6.39)

## Discussion

### Learning faster interceptions

Through the 10 learning blocks on five different days, participants learned to change their pattern of steering in the interception task. Differences between the old and the new pattern of steering were clearly revealed in participants’ interception duration, turning adjustment, target-heading angle, and the target’s bearing angle. In addition, through extensive learning, participants’ performance appeared to have reached a ceiling. First, the statistical analysis confirmed that participants produced the interception paths with comparable durations in the last two learning blocks (Fig. 4b). Second, participants’ interception paths changed little and almost overlapped one another in the last several learning blocks (see Fig. 4a).

In the first learning block, participants’ interception paths appeared clearly curved; the absolute rates of the target-heading angle in the late interception period were significantly lower than those of the target’s bearing angle. These results provide evidence that, before the extensive learning, participants’ interceptive steering is better described by the CTH strategy. This finding is consistent with our previous studies (Zhao, et al., 2019, 2021). In contrast, in the last learning block, participants’ interception paths appeared very close to linear and the absolute rates of the target-heading and the target’s bearing angle in the late interception period were not significantly different. Therefore, based on these results alone, participants’ interception strategies after learning were consistent with both the CTH and CB strategy, potentially allowing for the possibility that participants had learned the CB strategy and used it to guide their interception.

In theory, the CB strategy could have been learned based on the availability of the required information in the textured-ground environment. In this environment, the textured ground plane and the surrounding background landscape image were fixed within each trial. Thus, a stable allocentric reference frame could be established from the visual information. Additionally, during the interception task, the proprioceptive information from maneuvers of the steering wheel was available to participants. Thus, in theory, it could have been possible that the proprioceptive information during steering became sufficient to maintain a stable allocentric reference frame after extensive learning.

### Was the CB strategy learned?

If participants learned to use the CB strategy by utilizing the visual information about allocentric reference frames, they would be able to use it also in the textured-ground environment in the test session, but not in the green-ground environment, as this environment does not provide visual information about an allocentric reference frame. Thus, the learned interception behavior should be present in the textured-ground environment but absent in the green-ground environment, in the test session. If instead participants had learned to use the CB strategy based on proprioceptive information about allocentric reference frames, they would be able to use this strategy in both the textured-ground and the green-ground environment in the test session. Thus, the learned interception behavior should be present in both environments in the test session. However, the learned pattern of the target’s bearing angle, i.e., the comparable absolute rates over the early and the late interception period, were not well maintained in either environment during the test session. Specifically, in some conditions, the bearing angles’ absolute rates of change in the late period were significantly higher than those during the early period.

While a recent study (Casanova et al., 2022) interpreted the interception behavior when manipulating allocentric information by moving the environment (Chardenon et al., 2004; Fajen & Warren, 2004) as providing evidence in favor of reliance on bearing-angle-based information, these studies are quite different from the present study in terms of the task used and the manipulation of visual information. In particular, speed control tasks (Chardenon et al., 2004) may not allow distinguishing the involved strategies, as discussed above, and rotating the environment (Fajen & Warren, 2004) may lead to additional perceptual cue conflicts. Further research is needed to clarify the role of environment motion on locomotor interception.

In contrast, the learned pattern of the target-heading angle was well maintained between the learning and the test session. The peak-then-flat pattern of its absolute rate of change was observed across the two different sessions. Specifically, its absolute rates in the late interception period were always quite low and significantly lower than those in the early period. Moreover, this pattern was also observed during the first learning block, which measured participants’ baseline performance. Taken together, this suggests that this might be a general pattern in participants’ interceptions across different visual conditions and learning phases.

Further support for this interpretation comes from the fact that the absolute rates of change of the target-heading angle in the late interception period were either comparable or significantly lower than those of the target’s bearing angle in the test session. This suggests that the CTH strategy is a better account for interceptions in the test session. Taken together, the findings in the current study did not support the hypothesis that participants learned to abandon the CTH strategy and instead adopt the CB strategy to guide their locomotor interceptions. Instead, the results suggest that the CTH strategy can better account for participants’ interception behavior as a stable strategy across different visual conditions and learning phases.

One possible reason that the CB strategy was not learned and used by participants may be that the visual information about an allocentric reference frame is not reliable enough during learning. In the textured-ground environment, the surrounding background image was about 300 m away, which might be too far away to be sufficient to maintain a stable allocentric reference frame. The ground plane was covered with a random noise texture without any additional landmarks. Based on the texture, optic flow was available to participants even from the near ground. However, it has been suggested that optic flow alone is not sufficient to maintain a stable allocentric reference frame (Xu & Wallis, 2018). Concerning this possible reason, the question arises how the visual information about the allocentric reference frames could be strong enough for people to learn and use the CB strategy in the current task. It is an interesting question concerning our previous finding that people do not use the CB strategy in a similar interception task even when an apparent visual allocentric reference axis (i.e., a long straight wall) and dozens of landmarks (i.e., a dense arrangement of plants) are available in a similar open field environment (Zhao, et al., 2021). Further empirical studies are required to obtain a clear answer whether this is the exact reason.

That the CB strategy was not learned and used in the current study is consistent with the findings reported by Wallis and colleagues (Wallis, et al., 2002, 2007). They examined drivers’ performance in a lane changing task under conditions with or without visual information. In the condition without visual information, drivers did not change lanes successfully; instead, they usually showed a steering pattern that veers off the road with systematic errors in final heading. This finding suggests that, even though lane changing is a very basic and often practiced behavior for drivers, the proprioceptive information from maneuvers of the steering wheel alone is not sufficient for them to accurately maintain a stable allocentric reference frame. Therefore, this may be a possible reason that participants in the current study could not learn and use the CB strategy based on the proprioceptive information, even after the extensive learning.

### From funnel-like control to two-stage control

In the current study, our findings suggest that the CTH strategy is more consistent with participants’ interceptive steering than the CB strategy. Close inspections of details regarding the absolute rates of change of the target-heading angle may provide more implications concerning interception control. In the first learning block, after the initial adjustment (about the first10 bins in the left panel of Fig. 5b), the absolute rates of the target-heading angle gradually decreased until near the final moment of interception (at about the 45th bin in the left panel of Fig. 5b). The gradual decrease of the absolute rates of target-heading angle for the majority of the interception course is reminiscent of the “funnel-like type of control”. This type of control was originally proposed by Bootsma and colleagues to characterize the information-movement coupling in attacking forehand drives in table tennis (Bootsma & Van Wieringen, 1990; Bootsma et al., 1991). As it suggests, the strictness of the information-movement coupling increases as the action unfolds and reaches its maximum at the moment of ball-bat contact. According to this explanation, the funnel-like type of control is consistent with the change in the absolute rate of the target-heading angle in the first learning block in the current study. In the early period of interception, the CTH strategy might be loosely carried out to guide interception, resulting in relatively higher absolute rates of the target-heading angle; as the interception unfolded, the CTH strategy might be carried out strictly more and more, resulting in gradually decreasing absolute rates of the target-heading angle (see the left panel of Fig. 5b).

The funnel-like type of control suggests that strategies for action control are not always strictly carried out, especially in the early period of the action. A similar idea was also discussed in research on the visual control of goal-directed locomotion, such as door crossing (Camachon et al., 2007; Montagne et al., 2003) and moving-gap crossing (Chihak, et al., 2010). These studies demonstrate a prominent feature of two-stage control of the task. Specifically, people’s behavior in these locomotor tasks can be divided into two different stages. For example, when walking on a straight path to cross a pair of sliding doors ahead, people adjust their walking speed for successful crossing only in the last few seconds before crossing, not earlier during the approach (Montagne, et al., 2003).

Two-stage control was also proposed to account for the visual control of manual catching. In their experiment, de la Malla and López-Moliner (2015) asked participants to catch a ball approaching on a parabolic path with their hand. In the early period of catching, participants’ hand movements were more strongly influenced by the ball’s elevation angle and its derivative; in contrast, in the late period of catching, they were more strongly influenced by the ball’s looming information. Therefore, controls in the early and late catching periods seem qualitatively different based on different information available in the task.

In the current study, the peak-then-flat pattern of the absolute rates of the target-heading angle in the last learning block and the two test blocks clearly demonstrate the feature of a two-stage control of interceptive behavior (see Figs. 5b, 8). Specifically, the absolute rates rose and dropped quickly, resulting in a peak in the early interception period; then the rates were maintained at relatively low values in the late interception period. Moreover, the control of interception during the two stages is qualitatively different, with the late interception period showing behavior consistent with the CTH strategy, but the early interception period clearly deviating from the CTH strategy.

Was the CB strategy, instead of the CTH strategy, used in the early interception period during the last learning blocks? On the one hand, the absolute turning rates in the early interception period were significantly higher than those in the late interception period in these blocks (i.e., the peak-then-flat pattern). On the other hand, the CB strategy is not a good description of the interceptive steering in the late interception period based on the absolute rate of change of the bearing angle. Taken together, it seems not reasonable that participants could maintain an accurate allocentric reference frame and use the CB strategy with higher turning rates in the early interception period, but could not do it with lower turning rates in the late interception period.

Another possible explanation might be, that participants’ steering in the early interception period might be based on the information they received at the very beginning of each interception. Because they initially drove straight ahead without turning, their axis of motion can be regarded as a kind of allocentric reference axis. This information might be used within the CB strategy. Nevertheless, as they moved straight ahead, the target’s bearing angle was the same as the target-heading angle, very similarly to interception tasks along a straight path in which only the speed can be adjusted. In light of parsimony of explanations and based on the results of the control over the whole interception course, the target-heading angle might be a better explanation over the target’s bearing angle, including the steering in the early interception period.

Since the target-heading angle may play an important role in both the early and late interception periods, it is important to clarify the information from which it can be derived. As the car was always visible with its front pointing in the current heading direction in the two virtual environments, the target-heading angle could be derived as the angle between the target and the car’s anteroposterior axis. Moreover, in the textured-ground environment, optic flow can be used to specify participants’ heading direction (Bruggeman et al., 2007; Li & Cheng, 2011, 2013; Warren et al., 2001), based on which, the target-heading angle can be derived.

### Possible underlying mechanisms

In this paper, we have focused on behavioral evidence for and against proposed interception strategies based on bringing angular variables to a constant value. While participants in our experiment adopted a characteristic interception behavior that can be described succinctly as a two-stage control strategy, the underlying mechanisms of how such behavior comes about are not clear. Specifically, a theoretical account of what drives behavior in the first phase of the trial, which is not neatly described by any of the strategies based on angular variables, is still lacking.

One possible account could be based on the framework of behavioral dynamics proposed by Warren (2006). In this framework, an information-based approach to perception is integrated with a dynamical system approach to action. Based on this framework, Fajen and Warren (2007) proposed dynamics models to account for human interceptive steering. Their CB model was develop based on the CB strategy and the CTH model based on the CTH strategy. In the current study, participants’ interceptive steering was more consistent with the CTH strategy, at least in the late interception period, rather than the CB strategy. Nevertheless, Fajen and Warren’s (2007) CTH model, which nulls the change in the target-heading angle, cannot explain the increase in the target-heading angle in the early interception period in the current study. Therefore, it is still an open question how the current findings are explained based on the framework of behavioral dynamics.

Recently Bootsma and colleagues reported reversals in participants’ moving direction when they steered to intercept a moving target in virtual environments (Casanova, et al., 2022; Ceyte, et al., 2021). For example, actors first turn to the left side of the initial moving direction axis then turn to the right side of the axis. Upon this observation, they proposed that locomotor interception can be visually guided based on nulling fractional-order change in the target’s bearing angle (Casanova, et al., 2022). However, as with interception strategies based on integer-order derivatives, the mechanisms underlying behavior that can be described as nulling fractional-order derivatives are unclear. Moreover, this account, involving the target’s bearing angle, seems not able to explain participants' interceptions in the green-ground environment where information about an allocentric reference direction was missing, making it difficult to derive the target’s bearing angle.

Another possible account could be based on optimal stochastic control theory. Based on this theory, Belousov, Neumann, Rothkopf, and Peters (2016) proposed a computational model for locomotor interception of fly balls. Crucially, the model considers the agent’s sensory uncertainty, internal movement prediction uncertainty, locomotor control variability, as well as sensory delays. Although the model does not represent or calculate angular quantities such as the bearing angle or the target-heading angle explicitly, the optimal strategy under certain task conditions results in trajectories along which these angular quantities may stay constant. Thus, an outside observer would summarize the strategy as keeping angular quantities constant. Currently, it is still an open question how well this model can account for interceptive steering in different tasks including the one considered in the current experiments.

Taken together, future research both empirical and theoretical is required to explain, how human interception behavior is controlled and how the observed trajectories and dynamics come about.

### Limitations of current study

In the current study, participants controlled only the car’s heading direction but not its speed in the virtual environments, because this constraint on control can effectively help participants avoid motion sickness due to a conflict between visual and vestibular information (for motion sickness, see Bos et al., 2008). In contrast, in everyday driving, people control both a car’s heading direction and its speed in a real environment. Therefore, it cannot be excluded that people adopt another strategy when they perform locomotor interception under the latter circumstance.

In addition, in the current study, participants sat at the center of the car between the two front seats of the car for a better view of the environment. However, in daily driving, people sit at the driver’s seat by one side. The two sitting positions both allow people to derive the target-heading angle based on the information mentioned above, i.e., the car’s anteroposterior axis or optic flow. Therefore, it is possible that the two-stage control based on the target-heading angle is also used by drivers under real circumstances. Nevertheless, since the driver’s view may be blocked by the A-pillar of the car under real circumstances, the actual value of the target-heading angle during interception may be changed so that the target is not blocked by the A-pillar.

## Conclusion

Participants learned to intercept a moving target in virtual environments more efficiently across extensive training, by changing their steering patterns. While before learning they were following the CTH strategy for interception, they learned to adopt a two-stage control by first increasing the magnitude of the target-heading angle and subsequently following the CTH strategy. The learned pattern of the target-heading angle transferred to new target speeds, target path, and even a new environment, which did not provide visual information about an allocentric reference frame. By contrast, the learned pattern of the target’s bearing angle did not transfer well to the new conditions. Taken together, participants’ behavior was succinctly described by learning a two-stage control based on the target-heading angle for faster interception.

## Data Availability

The datasets generated and analyzed during the current study are available in the OSF repository, [https://osf.io/r6c79/?view_only=60dca45da21445ca8e045573ec7f6c02].

## Footnotes

1 The CTH and the CB strategies are equivalent to guide interception in the speed-control interception task. We use the CTH strategy to refer to both in this section.
